# Trajectories of Insomnia in Adults After Traumatic Brain Injury

**DOI:** 10.1001/jamanetworkopen.2021.45310

**Published:** 2022-01-26

**Authors:** Emerson M. Wickwire, Jennifer S. Albrecht, Vincent F. Capaldi, Sonia O. Jain, Raquel C. Gardner, J. Kent Werner, Pratik Mukherjee, Ashlee B. McKeon, Michael T. Smith, Joseph T. Giacino, Lindsay D. Nelson, Scott G. Williams, Jacob Collen, Xiaoying Sun, David M. Schnyer, Amy J. Markowitz, Geoffrey T. Manley, Andrew D. Krystal

**Affiliations:** 1 Department of Psychiatry, University of Maryland School of Medicine, Baltimore; 2 Sleep Disorders Center, Division of Pulmonary and Critical Care Medicine, Department of Medicine, University of Maryland School of Medicine, Baltimore; 3 Department of Epidemiology and Public Health, University of Maryland School of Medicine, Baltimore; 4 Center for Military Psychiatry and Neuroscience, Walter Reed Army Institute of Research, Silver Spring, Maryland; 5 Department of Medicine, Uniformed Services University of the Health Sciences, Bethesda, Maryland; 6 Biostatistics Research Center, Herbert Wertheim School of Public Health and Human Longevity Science, University of California, San Diego; 7 Department of Neurology, University of California, San Francisco; 8 Department of Neurology, Uniformed Services University of the Health Sciences, Bethesda, Maryland; 9 Department of Neurology, The Johns Hopkins University, Baltimore, Maryland; 10 Department of Radiology, School of Medicine, University of California, San Francisco; 11 Division of Behavioral Medicine, Department of Psychiatry, Johns Hopkins University School of Medicine, Baltimore, Maryland; 12 Department of Physical Medicine and Rehabilitation, Harvard Medical School, Boston, Massachusetts; 13 Spaulding Rehabilitation Hospital, Charlestown, Massachusetts; 14 Department of Neurosurgery, Medical College of Wisconsin, Milwaukee; 15 Department of Neurology, Medical College of Wisconsin, Milwaukee; 16 Department of Medicine, Fort Belvoir Community Hospital, Fort Belvoir, Virginia; 17 Department of Psychiatry, Uniformed Services University of the Health Sciences, Bethesda, Maryland; 18 Sleep Disorders Center, Department of Medicine, Walter Reed National Military Medical Center, Bethesda, Maryland; 19 Department of Psychology, University of Texas at Austin; 20 Department of Epidemiology and Biostatistics, School of Medicine, University of California, San Francisco; 21 Brain and Spinal Injury Center, University of California, San Francisco; 22 Department of Neurosurgery, University of California, San Francisco; 23 Department of Psychiatry and Behavioral Sciences, University of California, San Francisco; 24 Weill Institute for Neurosciences, University of California, San Francisco

## Abstract

**Importance:**

Insomnia is common after traumatic brain injury (TBI) and contributes to morbidity and long-term sequelae.

**Objective:**

To identify unique trajectories of insomnia in the 12 months after TBI.

**Design, Setting, and Participants:**

In this prospective cohort study, latent class mixed models (LCMMs) were used to model insomnia trajectories over time and to classify participants into distinct profile groups. Data from the Transforming Research and Clinical Knowledge in Traumatic Brain Injury (TRACK-TBI) study, a longitudinal, multisite, observational study, were uploaded to the Federal Interagency Traumatic Brain Injury Repository (FITBIR) database. Participants were enrolled at 1 of 18 participating level I trauma centers and enrolled within 24 hours of TBI injury. Additional data were obtained directly from the TRACK-TBI investigators that will be uploaded to FITBIR in the future. Data were collected from February 26, 2014, to August 8, 2018, and analyzed from July 1, 2020, to November 15, 2021.

**Exposures:**

Traumatic brain injury.

**Main Outcomes and Measures:**

Insomnia Severity Index assessed serially at 2 weeks and 3, 6, and 12 months thereafter.

**Results:**

The final sample included 2022 participants (1377 [68.1%] men; mean [SD] age, 40.1 [17.2] years) from the FITBIR database and the TRACK-TBI study. The data were best fit by a 5-class LCMM. Of these participants, 1245 (61.6%) reported persistent mild insomnia symptoms (class 1); 627 (31.0%) initially reported mild insomnia symptoms that resolved over time (class 2); 91 (4.5%) reported persistent severe insomnia symptoms (class 3); 44 (2.2%) initially reported severe insomnia symptoms that resolved by 12 months (class 4); and 15 (0.7%) initially reported no insomnia symptoms but had severe symptoms by 12 months (class 5). In a multinomial logistic regression model, several factors significantly associated with insomnia trajectory class membership were identified, including female sex (odds ratio [OR], 1.65 [95% CI, 1.02-2.66]), Black race (OR, 2.36 [95% CI, 1.39-4.01]), history of psychiatric illness (OR, 2.21 [95% CI, 1.35-3.60]), and findings consistent with intracranial injury on computed tomography (OR, 0.36 [95% CI, 0.20-0.65]) when comparing class 3 with class 1.

**Conclusions and Relevance:**

These results suggest important heterogeneity in the course of insomnia after TBI in adults. More work is needed to identify outcomes associated with these insomnia trajectory class subgroups and to identify optimal subgroup-specific treatment approaches.

## Key Points

**Question:**

What is the natural history of insomnia in the 12 months after traumatic brain injury (TBI)?

**Findings:**

In this analysis of 2022 adults from the large cohort study Transforming Research and Clinical Knowledge in Traumatic Brain Injury, insomnia was common during the 12 months after TBI. Five trajectory classes of insomnia were identified, and baseline factors including sex, race and ethnicity, history of TBI, psychiatric history, and computed tomography status were identified as significantly associated with insomnia trajectory class membership.

**Meaning:**

These findings suggest that insomnia is common after TBI and should be assessed early in recovery.

## Teaser

This cohort study of 2022 adults with traumatic brain injury identifies unique trajectories of insomnia during the 12 months after injury based on serial assessment using standardized measures.

## Introduction

Traumatic brain injury (TBI) represents a major public health burden by any standard. Each year in the US alone, at least 2.5 million adults seek medical care for TBI.^1^ Traumatic brain injury also incurs substantial health-related and economic costs, including increased risk of fatigue, depression, posttraumatic stress disorder, chronic pain, impaired cognition, diminished quality of life, and other adverse outcomes.^2,3,4,5,6^ Insomnia, which is defined as difficulty initiating or maintaining sleep with associated daytime impairment, is among the most common complaints after TBI. Meta-analytic findings suggest an insomnia prevalence rate of 29% among adults with a history of TBI of any severity.^7^ This rate is approximately twice the prevalence of insomnia among adults in the general population.

Insomnia can develop during the acute (0-7 days), subacute (8-90 days), or chronic (>90 days) phase after TBI.^8,9^ Not only can TBI disturb sleep, but there is evidence that poor sleep is associated with neurodegeneration,^10^ independently contributing to morbidity and long-term sequelae of TBI.^8,9,11^ Evidence from epidemiological, clinical, and experimental non-TBI cohorts demonstrates that insomnia worsens outcomes in patients with depression,^12^ posttraumatic stress disorder,^13^ and chronic pain^14^ and impairs cognitive and functional performance.^15^ Insomnia can precede, exacerbate, and/or prolong each of these conditions, with a profound negative impact on health-related quality of life and increased economic costs, including use of health care services and disability.^16,17,18,19,20^ Furthermore, individuals with TBI report that symptoms of insomnia are among their most troubling problems.^21^ In part because it is highly treatable when it occurs in settings outside TBI, insomnia is increasingly recognized as a promising modifiable treatment target with the potential to improve rehabilitation and long-term functional outcomes after TBI.^8,9,22,23,24^

Studies of insomnia in TBI to date have been hampered by heterogeneous TBI samples, inconsistent operational definitions of insomnia, and highly variable follow-up durations, with most studies only assessing outcomes at 2 points in time, potentially masking variable courses of recovery.^8,9^ As a result, little is known about the natural history of insomnia after TBI. To address this gap in knowledge, the present study aimed to identify clinically and biologically relevant subgroups of patients with post-TBI insomnia, leveraging the rich, multicenter Transforming Research and Clinical Knowledge in Traumatic Brain Injury (TRACK-TBI) cohort. By using advanced, data-driven analytic approaches, we sought to identify unique trajectories of insomnia during the 12 months after TBI based on serial assessment using standardized measures. We hypothesized that distinct trajectories of insomnia symptoms would emerge.

## Methods

### Participants

This cohort study adhered to the Strengthening the Reporting of Observational Studies in Epidemiology (STROBE) reporting guideline. Participants were identified via the Federal Interagency Traumatic Brain Injury Repository (FITBIR). FITBIR is an ongoing data repository including data from federally funded research projects in the US. Participants are identified only by a global unique identifier, and common data elements were collected based on established guidelines.^25^

Data were collected from February 26, 2014, to August 8, 2018. The study included participants in TRACK-TBI aged 17 years or older whose data were uploaded into the FITBIR database, supplemented with data obtained directly from the TRACK-TBI investigators that is scheduled to be uploaded to FITBIR in the future. TRACK-TBI is a longitudinal, multisite observational study.^25^ Race and ethnicity data, which were obtained via self report during clinical interviews, were collected because they have been associated with TBI outcomes. Race and ethnicity were established as 2 separate variables. Participants were enrolled at 1 of 18 participating level I trauma centers. Participants in TRACK-TBI were enrolled within 24 hours of TBI injury and completed serial assessments at 2 weeks and 3, 6, and 12 months thereafter. These assessments captured clinical, neuroimaging, and blood biomarkers to improve TBI classification and outcome assessments.^25^ TRACK-TBI procedures are described in detail elsewhere.^25,26^ Participants were included in the present trajectory analysis if they had at least 1 Insomnia Severity Index (ISI) measurement, which was administered at 2 weeks and 3, 6, and 12 months after injury. This study was determined to be non–human participant research by the institutional review board at the University of Maryland, Baltimore, and therefore did not require informed consent.

### Measures

Demographic and medical history information was obtained at enrollment in TRACK-TBI within 24 hours after injury. The ISI is a 7-item self-report measure that assesses nighttime and daytime symptoms of insomnia with individual item scores ranging from 0 to 4 and total scores ranging from 0 to 28. Based on this total score, 2 systems of insomnia classification have been used. Morin et al^27^ identified an ISI score of greater than 10 as the optimal cut point to identify likely insomnia cases (eg, for prevalence estimates). In addition, the ISI total score can be further classified into 4 ordinal levels of insomnia severity: no clinically significant insomnia (0-7) and mild (8-14), moderate (15-21), and severe insomnia (22-28).^28^ The ISI has demonstrated excellent psychometric properties and sensitivity to insomnia treatment-related change^27,29^ and has been used in numerous prior studies of TBI.

### Statistical Analysis

Data were analyzed from July 1, 2020, to November 15, 2021. Descriptive summaries were used to describe the study cohort. Unconditional latent class mixed models (LCMMs) for ordinal data were used to model ISI trajectories over time and to classify participants into distinct latent trajectory classes.^30,31^ We fitted models with 1 to 7 classes and selected the best model based on assessment of the Akaike information criterion (AIC), bayesian information criterion, log likelihood, and consideration of clinical relevance (eTable in Supplement 1). The posterior probabilities of membership for each participant in each trajectory class were estimated using the maximum probability assignment rule of Strauss et al.^32^ Each individual participant was assigned to the class with their highest probability of membership. Spaghetti plots (eFigure 1 in Supplement 1) were used to visualize individual trajectories within each class category, and a river plot (eFigure 2 in Supplement 1) was used to visualize the flow of patients between classes in each successive model to determine clinical relevance and face validity of the data-driven trajectory classes, as described in detail previously.^33^

After model selection, we compared the demographic and baseline clinical characteristics between the latent class groups. Between-group differences were evaluated using a Fisher exact test for categorical variables and a Kruskal-Wallis test for continuous variables. Variables significantly associated with trajectory group membership in bivariate analysis were included in a multinomial logistic regression model to assess the independent association of baseline factors with latent trajectory class membership, with the largest class (consistent mild insomnia) as the reference group.

Analyses were performed using statistical software R, version 3.6.1 (R Project for Statistical Computing). The lcmm package was used for the LCMM analysis. Two-sided *P* < .05 indicated statistical significance.

## Results

### Participant Characteristics

Table 1 presents demographic and clinical characteristics of TRACK-TBI participants with at least 1 ISI assessment overall by insomnia trajectory class. The mean (SD) age was 40.1 (17.2) years; 1377 participants [68.1%]) were men and 645 (31.9%) were women. Race was available for 2012 participants; ethnicity was available for 2014 participants. Among the 2022 participants, 330 of 2012 (16.4%) were Black; 413 of 2014 (20.5%) were Hispanic, 1561 of 2012 (77.6%) were White, and 121 of 2012 (6.0%) were other (including Alaska Native or Inuit, American Indian, Asian, and Native Hawaiian or Other Pacific Islander). Among those with data available, the most common cause of TBI was a motor vehicle crash (1156 of 2015 [57.4%]). Most participants had mild TBI, defined by a Glasgow Coma Scale score of 13 to 15 (1720 of 1988 [86.5%]). More than one-fifth of participants (451 of 2022 [22.3%]) reported a history of any psychiatric disorder, including depression (299 of 2022 [14.8%]) and anxiety (252 of 2022 [12.5%]).

**Table 1.** Baseline Characteristics of TRACK-TBI Participants With 1 or More ISI Measurement

Characteristic	ISI trajectory class^a^						*P* value^b^
	All (N = 2022)	Class 1 (n = 1245)	Class 2 (n = 627)	Class 3 (n = 91)	Class 4 (n = 44)	Class 5 (n = 15)	
Age, mean (SD), y	40.1 (17.2)	39.5 (16.5)	41.5 (19.1)	40.6 (14.1)	39.7 (13.6)	37.7 (13.6)	.45
Sex							
Men	1377/2022 (68.1)	863/1245 (69.3)	431/627 (68.7)	49/91 (53.8)	26/44 (59.1)	8/15 (53.3)	.02
Women	645/2022 (31.9)	382/1245 (30.7)	196/627 (31.3)	42/91 (46.1)	18/44 (40.9)	7/15 (46.7)	
Race							
Black	330/2012 (16.4)	208/1239 (16.8)	71/624 (11.4)	33/90 (36.7)	14/44 (31.8)	4/15 (26.7)	<.001
White	1561/2012 (77.6)	959/1239 (77.4)	506/624 (81.1)	56/90 (62.2)	29/44 (65.9)	11/15 (73.3)	
Other^c^	121/2012 (6.0)	72/1239 (5.8)	47/624 (7.5)	1/90 (1.1)	1/44 (2.3)	0	
Hispanic ethnicity	413/2014 (20.5)	281/1241 (22.6)	106/623 (17.0)	10/91 (11.0)	13/44 (29.5)	3/15 (20.0)	.003
Educational level, mean (SD), y^d^	13.5 (2.9)	13.2 (2.9)	14.2 (2.8)	13.0 (2.3)	12.9 (2.8)	12.8 (2.5)	<.001
Injury mechanism							
Car crash	1156/2015 (57.4)	707/1241 (57.0)	355/625 (56.8)	54/91 (59.3)	31/43 (72.1)	9/15 (60.0)	.02
Fall	526/2015 (26.1)	330/1241 (26.6)	170/625 (27.2)	17/91 (18.7)	7/43 (16.3)	2/15 (13.3)	
Assault	140/2015 (6.9)	98/1241 (7.9)	28/625 (4.5)	9/91 (9.9)	2/43 (4.7)	3/15 (20.0)	
Other	193/2015 (9.6)	106/1241 (8.5)	72/625 (11.5)	11/91 (12.1)	3/43 (7.0)	1/15 (6.7)	
Glasgow Coma Scale in ED							
13-15	1720/1988 (86.5)	1057/1225 (86.3)	522/614 (85.0)	85/91 (93.4)	43/44 (97.7)	13/14 (92.9)	.12
9-12	78/1988 (3.9)	56/1225 (4.6)	21/614 (3.4)	1/91 (1.1)	0	0	
3-8	190/1988 (9.6)	112/1225 (9.1)	71/614 (11.6)	5/91 (5.5)	1/44 (2.3)	1/14 (7.1)	
AIS head score, mean (SD)^e^	2.4 (1.4)	2.4 (1.5)	2.5 (1.4)	1.7 (1.3)	1.6 (1.0)	2.8 (1.2)	<.001
Computed tomographic finding							
Positive	836/1957 (42.7)	510/1196 (42.6)	293/614 (47.7)	18/89 (20.2)	8/43 (18.6)	7/15 (46.7)	<.001
Negative	1121/1957 (57.3)	686/1196 (57.4)	321/614 (52.3)	71/89 (79.8)	35/43 (81.4)	8/15 (53.3)	
History of TBI	604/1986 (30.4)	373/1218 (30.6)	168/619 (27.1)	42/90 (46.7)	14/44 (31.8)	7/15 (46.7)	.003
Comorbid conditions							
Any psychiatric history	451/2022 (22.3)	306/1245 (24.6)	85/627 (13.6)	41/91 (45.1)	14/44 (31.8)	5/15 (33.3)	<.001
Anxiety	252/2022 (12.5)	170/1245 (13.7)	41/627 (6.5)	29/91 (31.9)	10/44 (22.7)	2/15 (13.3)	<.001
Depression	299/2022 (14.8)	207/1245 (16.6)	51/627 (8.1)	28/91 (30.8)	8/44 (18.2)	5/15 (33.3)	<.001
Diabetes	126/2022 (6.2)	79/1245 (6.3)	36/627 (5.7)	7/91 (7.7)	1/44 (2.3)	3/15 (20.0)	.19
Hyperlipidemia	121/2022 (6.0)	70/1245 (5.6)	44/627 (7.0)	4/91 (4.4)	7/44 (15.9)	2/15 (13.3)	.33
Hypertension	301/2022 (14.9)	176/1245 (14.1)	93/627 (14.8)	20/91 (22.0)	8/44 (18.2)	4/15 (26.7)	.15
Problem alcohol use	835/1950 (42.8)	518/1195 (43.4)	266/607 (43.8)	34/89 (38.2)	8/44 (18.2)	9/15 (60.0)	.008
Past 12-mo illicit drug use	497/1940 (25.6)	329/1190 (27.7)	128/601 (21.3)	29/90 (32.2)	7/44 (15.9)	4/15 (26.7)	.01
Sleep disorder	68/2022 (3.4)	41/1245 (3.3)	6/627 (1.0)	17/91 (18.7)	2/44 (4.5)	2/15 (13.3)	<.001
Sleep apnea	43/2022 (2.1)	28/1245 (2.2)	9/627 (1.4)	4/91 (4.4)	1/44 (2.3)	1/15 (6.7)	.14

Abbreviations: AIS, Abbreviated Injury Scale; ED, emergency department; ISI, Insomnia Severity Index; TBI, traumatic brain injury; TRACK-TBI, Transforming Research and Clinical Knowledge in Traumatic Brain Injury.

^a^ All participants were 17 years or older. Class 1 indicates mild insomnia symptoms that did not change over time; class 2, mild insomnia symptoms that resolved over time; class 3, consistently severe insomnia symptoms; class 4, initially severe insomnia symptoms that resolved by 12 months; and class 5, no initial reports of insomnia symptoms but severe symptoms at 12 months. Unless otherwise indicated, data are expressed as number/total number (%) of participants. Percentages have been rounded and may not total 100.

^b^ Calculated using a Fisher exact test for categorical variables and a Kruskal-Wallis test for continuous variables.

^c^ Includes Alaska Native or Inuit, American Indian, Asian, and Native Hawaiian or Other Pacific Islander.

^d^ Includes 1971 participants.

^e^ Available only for participants who were hospitalized (n = 1541).

Relative to TRACK-TBI participants without an ISI assessment (n = 530) not included in this analysis, the 2022 participants in the study sample were younger (mean [SD] age, 40.1 [17.2] vs 46.2 [18.5] years; *P* < .001) and less likely to be male (1377 [68.1%] vs 390 [73.6%]; *P* = .02). They were less severely injured; only 190 of 1988 (9.6%) had severe TBI (Glasgow Coma Scale score, 3-8) compared with 172 (34.6%) of those without an ISI assessment (*P* < .001).

### Insomnia Severity

**Figure 1.**
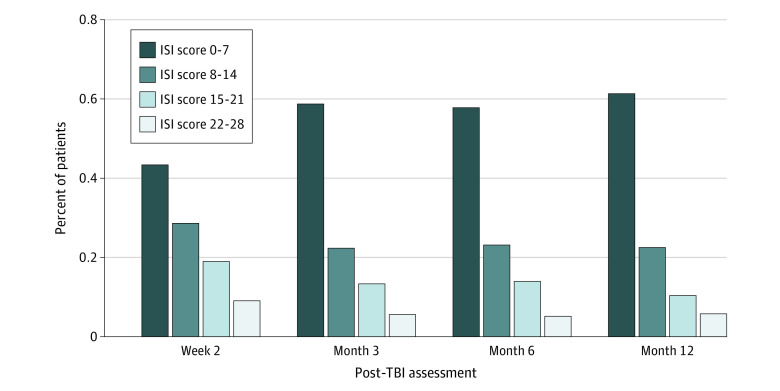
Insomnia Severity Categories by Time Since Injury Includes 2022 participants 17 years or older from the Transforming Research and Clinical Knowledge in Traumatic Brain Injury (TBI) study. ISI indicates Insomnia Severity Index, with higher scores indicating greater severity.

A total of 1114 patients (55.0%) had complete ISI scores at all 4 assessments; 462 (22.8%) had missing data at 1 assessment and 446 (22.0%) had missing data at 2 or 3 assessments. Based on the threshold for likely insomnia (ISI score >10) at 2 weeks after TBI, 740 of 1709 participants (43.3%) had likely insomnia disorder. By the 12-month visit, only 418 of 1502 (27.8%) continued to have likely insomnia disorder. In addition, Figure 1 presents the percentage of participants in each ISI category at each time point. At the 2-week assessment, 479 (28.0%) participants had moderate or severe insomnia symptoms, and only 741 (43.4%) experienced no clinically significant insomnia symptoms. By the 12-month visit, only 243 (16.2%) had moderate or severe insomnia symptoms, and 921 (61.3%) reported no clinically significant insomnia symptoms.

### Trajectories of Insomnia

**Figure 2.**
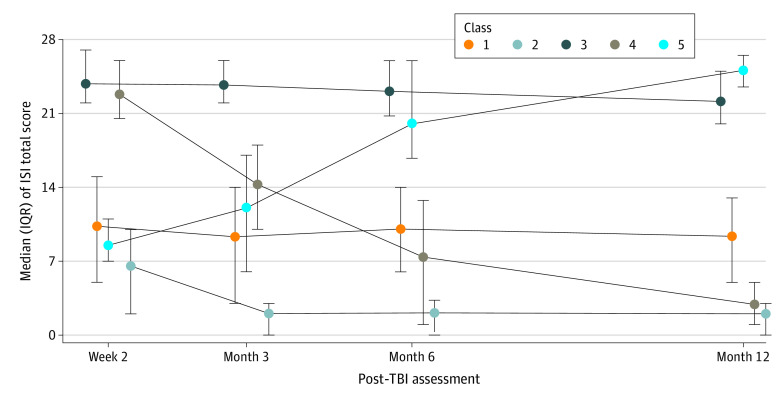
Five-Class Model of Trajectories of Insomnia After Traumatic Brain Injury (TBI) Includes 2022 participants 17 years or older from the Transforming Research and Clinical Knowledge in Traumatic Brain Injury study. Five classes of insomnia trajectory were identified: mild insomnia symptoms that did not change over time (class 1); mild insomnia symptoms that resolved over time (class 2); consistently severe insomnia symptoms (class 3); initially severe insomnia symptoms that resolved by 12 months (class 4); and no initial reports of insomnia symptoms followed by severe symptoms at 12 months (class 5). ISI indicates Insomnia Severity Index.

The data were best fit by 4- and 5-class LCMMs based on model fit parameters (eg, 4-class AIC, 12634.91; 5-class AIC, 12636.76; and other model AICs, 12639.41-12778.91). Although the AIC reached absolute minimum for the 4-class model, the 5-class LCMM was selected based on clinical relevance (ie, based on clinical experience), posterior membership class probabilities, and review and discussion of plots. Figure 2 depicts the 5-class model. Class 1 (1245 of 2022 [61.6%]) reported mild insomnia symptoms that did not change over time. Class 2 (627 of 2022 [31.0%]) initially reported mild insomnia symptoms that resolved over time. Class 3 (91 of 2022 [4.5%]) reported consistently severe insomnia symptoms. Class 4 initially reported severe insomnia symptoms that resolved by 12 months (44 of 2022 [2.2%]); class 5 initially reported no insomnia symptoms but reported severe symptoms at 12 months (15 of 2022 [0.7%]).

### Association Between Demographic and Clinical Characteristics and Insomnia Trajectory Class

Multiple differences in demographic and clinical characteristics were observed across insomnia trajectory classes in initial analysis (Table 1). Results from our multinomial regression model are presented in Table 2. Relative to individuals in class 1 (consistent mild insomnia) and compared with all other racial and ethnic groups, Black individuals had decreased odds of being in class 2 (initial mild symptoms that resolve) (odds ratio [OR], 0.64 [95% CI, 0.47-0.89]) and increased odds of being in class 3 (consistent severe insomnia) (OR, 2.36 [95% CI, 1.39-4.01]). Similarly, relative to individuals with no psychiatric history, those with any psychiatric history had decreased odds of being in class 2 (OR, 0.38 [95% CI, 0. 28-0.51]) and increased odds of being in class 3 (OR, 2.21 [95% CI, 1.35-3.60]). Relative to class 1 and compared with having normal findings on a computed tomographic scan, a computed tomographic scan with evidence of intracranial injury was associated with decreased odds of being in class 3 (OR, 0.36 [95% CI, 0.20-0.65]) and class 4 (initial severe symptoms that resolve) (OR, 0.40 [95% CI, 0.18-0.88]). Female sex was associated with increased odds of being in class 3 (OR, 1.65 [95% CI, 1.02-2.66]).

**Table 2.** Independent Associations of Demographic and Clinical Characteristics With Insomnia Trajectory Class Membership^a^

Characteristic	OR (95% CI)			
	Class 2 (n = 627)	Class 3 (n = 91)	Class 4 (n = 44)	Class 5 (n = 15)
Years of education	1.13 (1.09-1.18)	0.96 (0.88-1.06)	1.02 (0.91-1.16)	0.95 (0.77-1.15)
Female vs male sex	1.14 (0.90-1.43)	1.65 (1.02-2.66)	1.24 (0.64-2.40)	2.65 (0.87-8.05)
Black vs all other racial and ethnic groups	0.64 (0.47-0.89)	2.36 (1.39-4.01)	2.19 (0.99-4.82)	1.92 (0.53-6.93)
Assault vs other cause of injury	0.69 (0.43-1.10)	1.46 (0.65-3.29)	0.33 (0.04-2.47)	2.70 (0.67-10.97)
Prior TBI vs none	0.89 (0.71-1.13)	1.74 (1.09-2.79)	1.16 (0.59-2.30)	2.02 (0.69-5.84)
Any psychiatric history vs none	0.38 (0.28-0.51)	2.21 (1.35-3.60)	1.36 (0.65-2.85)	1.16 (0.36-3.72)
Problem alcohol use vs none	1.01 (0.81-1.24)	0.93 (0.58-1.48)	0.30 (0.13-0.68)	2.01 (0.69-5.84)
Computed tomography findings positive vs negative	1.17 (0.94-1.44)	0.36 (0.20-0.65)	0.40 (0.18-0.88)	1.39 (0.48-4.05)

Abbreviations: OR, odds ratio; TBI, traumatic brain injury.

^a^ Class 1 (n = 1245) (mild insomnia symptoms that did not change over time) was the reference category. Class 2 indicated mild insomnia symptoms that resolved over time; class 3, consistently severe insomnia symptoms; class 4, initially severe insomnia symptoms that resolved by 12 months; and class 5, no initial reports of insomnia symptoms but severe symptoms at 12 months.

## Discussion

In this study evaluating post-TBI insomnia longitudinally during 12 months in a large sample of adult patients with primarily mild TBI (Glasgow Coma Scale score, 13-15) and using a well-accepted measure of insomnia symptoms (the ISI), we observed elevated rates of insomnia at all time points. Specifically, the prevalence of likely clinical insomnia was 43.3% at 2 weeks, which decreased to 27.8% at the 12-month follow-up, compared with 10% among adults in the general adult population.^34^ Furthermore, we used advanced, data-driven trajectory modeling approaches^33^ and identified 5 novel trajectories of insomnia after TBI that have not been reported previously. A number of noteworthy findings emerged with respect to the prevalence and severity of insomnia as well as the insomnia trajectory subgroups themselves, which represent important contributions to our understanding of the natural history of insomnia in patients with TBI.

Our finding that insomnia is common after TBI is consistent with those of prior studies,^7^ and validated categorical ISI scores also suggest a markedly high prevalence of moderate and severe insomnia at 2 weeks (28.0%) and 12 months (16.2%), substantially exceeding the prevalence of all clinical insomnia in the general adult population. From a prognostic perspective, although the prevalence of insomnia decreases during the 12 months after TBI, insomnia (including moderate and severe insomnia) remains very common at 1 year after TBI.

Our results also provide novel insight into the natural history of insomnia after TBI. Others have considered the natural history of insomnia in the general population,^35^ among military service members,^36^ or during the transition from acute to chronic insomnia.^37^ By contrast, using data-driven analytic approaches,^33^ we identified 5 latent class trajectory groups of insomnia after TBI. Among our study participants, 61.6% reported persistent mild insomnia (ISI score, 8-14), and a considerable portion of these individuals are likely to benefit from treatment. An additional 31.0% of participants initially reported mild insomnia that resolved over time. Of particular interest, 3 subgroups representing 7.4% of our sample reported severe insomnia symptoms. One of these groups included individuals representing 2.2% of the sample who initially reported severe insomnia symptoms that resolved over time. Such individuals are likely to be substantially affected by their sleep disturbance and should be considered for intervention as soon as possible after TBI to mitigate the impact of their insomnia on recovery during the early post-TBI period. The reasons why insomnia symptoms improved in these individuals are unclear. We lack data on any treatments that these individuals might have received after TBI and whether observed changes in insomnia symptoms were due to treatments or simply reflect the natural course of insomnia after TBI in these individuals.

Two trajectory classes were notable for having significant symptoms of insomnia that did not resolve during the first year after TBI. Class 3, constituting 4.5% of the sample, had severe insomnia symptoms that persisted throughout the study period. Class 5 was composed of relatively few individuals (0.7%) who did not have insomnia initially but developed severe insomnia within 12 months after injury. Based on their early symptoms, those with persistent severe insomnia could be identified and targeted for insomnia-focused treatment and support in the early post-TBI period. However, the reasons their insomnia symptoms persisted remain unknown. We are without data on treatments received after TBI that could inform whether insomnia persistence represents a lack of treatment or treatment failure, which itself might provide an indication of what types of treatments could show efficacy. In addition, the results of our multinomial regression model found that female sex, Black race, history of TBI, and psychiatric history were all associated with severe persistent insomnia. Further work is needed to determine mechanisms underlying these associations and how to personalize insomnia treatments based on these and other patient characteristics.

### Strengths and Limitations

The strengths of this study include a large, well-characterized sample from leading TBI research centers throughout the US and robust longitudinal data on insomnia symptoms collected using a well-validated instrument. Detailed information on demographic, clinical, and injury-related variables was available, permitting an exploration of characteristics of participants in each insomnia trajectory group. The research question was novel and leveraged an established analytic approach (LCMM) that others have used to identify trajectories in other outcomes variables within the TRACK-TBI data.^33^ Our trajectory-based approach provides important insight that would have been unattainable via cross-sectional procedures. As an example, our approach highlights the likely very different biopsychosocial causes of insomnia across trajectory groups. We intend to explore these differences in future work.

We also acknowledge several limitations of this study. First, TRACK-TBI included only patients presenting within 24 hours of injury to level I trauma centers. Thus, generalizability of these results to individuals with milder injuries presenting to nontrauma centers is unknown. Certainly, our trajectory findings should be replicated in other large samples of patients with TBI. Similarly, relative to participants without an ISI assessment, those with an ISI (ie, those included in this analysis) were less severely injured. Given observed differences in TBI severity across insomnia trajectory groups, the impact of these differences is unclear. However, it is notable that TBI injury severity was positively associated with insomnia data missingness, further contributing to an underrepresentation of moderate and severe TBI and limiting our ability to assess the association between TBI severity and trajectory group status. At minimum, future research should seek to replicate current results among individuals with more severe TBI. Nonetheless, most TBI is mild, suggesting that these results may generalize well to other populations with TBI. Another limitation is that this study lacked objective measurement of sleep, such as actigraphy (a validated proxy for sleep) and polysomnography. Expert consensus has supported multimethod assessment of sleep after TBI, including subjective and objective measures.^8^ Third, more sensitive measurement of sleep and other health behaviors (eg, alcohol use) will enable more nuanced analysis of the associations between these variables and key TBI outcomes. Fourth, although our data-driven approach produced novel and important results, it is possible that we missed other clinically meaningful trajectory groups. Fifth, although Black race was associated with adverse insomnia outcomes, we were unable to assess the mechanisms of this result, such as structural racism or perceived discrimination. Last, and perhaps most important, our cohort study design precludes determination of causality. The extent to which TBI caused insomnia observed in our sample is unknown. One-fifth of participants in our sample reported a history of a psychiatric disorder, which is well known to increase risk for insomnia. Likewise, chronic pain and posttraumatic stress disorder are common after TBI and are known to worsen insomnia severity. These limitations should be addressed in future work to better advance understanding of insomnia trajectories after TBI, to identify individuals at risk for adverse insomnia outcomes, and to inform best approaches to subgroup-specific treatments. Building on our findings as a springboard to future research promises to advance TBI care toward a precision medicine paradigm in which support and treatment are targeted to achieve resolution of insomnia in patients with this sequela. Such research will also advance our ability to design future clinical trials in patients with TBI in a rational manner.

## Conclusions

To our knowledge, this cohort study represents the largest analysis of insomnia after TBI to date. The prevalence of insomnia was notably high, particularly for moderate and severe insomnia. Using data-driven analytic approaches, we identified 5 distinct trajectories of insomnia in the first year after TBI. Analysis of these subgroups generated a number of insights with the potential to inform clinical care and research. In addition to persistent mild insomnia for most of the participants, 3 trajectory subgroups totaling 7.4% of the sample had severe insomnia, and for most of these individuals this insomnia did not resolve within 12 months after injury. Black race, female sex, pre-TBI psychiatric history, and prior TBI were associated with persistent severe insomnia. However, more work is needed to identify mechanisms underlying these associations, to understand the impact of insomnia trajectory group status on TBI outcomes, and to identify optimal subgroup-specific treatment approaches.

## Notes

Supplement 1. **eTable.** Model Fit Parameters for Latent Class Mixture Models With 1-7 Classes **eFigure 1.** Randomly Selected Raw ISI Category Level Data From ≤10 Patients in Each Class **eFigure 2.** River Plot

Supplement 2.
Nonauthor Collaborators
