# Supplementary material for: Trajectories of Insomnia in Adults After Traumatic Brain Injury
Source: JAMA Netw Open. 2022 Jan 26;5(1):e2145310. doi: 10.1001/jamanetworkopen.2021.45310 (PMC8792888; doi:10.1001/jamanetworkopen.2021.45310)
Supplement: Supplement 1. — eTable. Model Fit Parameters for Latent Class Mixture Models With 1-7 Classes eFigure 1. Randomly Selected Raw ISI Category Level Data From ≤10 Patients in Each Class eFigure 2. River Plot [file jamanetwopen-e2145310-s001.pdf]

Supplementary Online Content

Wickwire EM, Albrecht JS, Capaldi VF II, et al; Transforming Research and Clinical Knowledge in Traumatic Brain Injury (TRACK-TBI) Investigators. Trajectories of insomnia in adults after traumatic brain injury. *JAMA Netw Open*. 2022;5(1):e2145310. doi:10.1001/jamanetworkopen.2021.45310

eTable. Model Fit Parameters for Latent Class Mixture Models With 1-7 Classes

eFigure 1. Randomly Selected Raw ISI Category Level Data From ≤10 Patients in Each Class

eFigure 2. River Plot

This supplementary material has been provided by the authors to give readers additional information about their work.

© 2022 Wickwire EM et al. *JAMA Network Open*.

eTable. Model Fit Parameters for Latent Class Mixture Models With 1-7 Classes

No of classes in model	loglik	AIC	BIC
1	-6384.455	12778.91	12806.97
2	-6318.492	12652.98	12697.88
3	-6310.203	12642.41	12704.14
4	-6303.454	12634.91	12713.47
5	-6301.381	12636.76	12732.16
6	-6299.704	12639.41	12751.65
7	-6299.139	12644.28	12773.35

Loglik=log likelihood. AIC=Akaike information criterion. BIC=Bayesian information criterion.

© 2022 Wickwire EM et al. *JAMA Network Open*.

eFigure 1. Randomly Selected Raw ISI Category Level Data From ≤10 Patients in Each Class

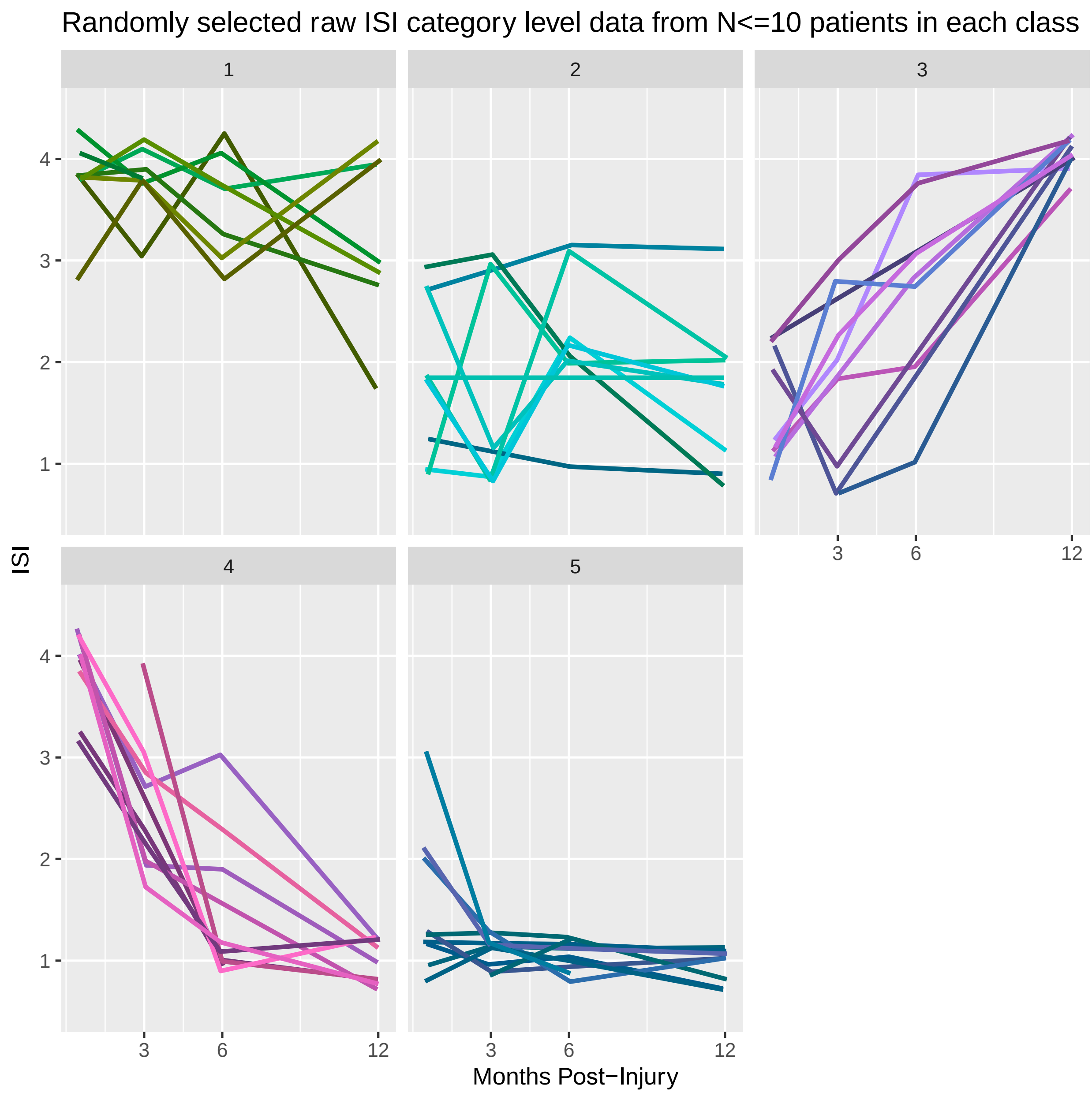

ISI=Insomnia Severity Index.

© 2022 Wickwire EM et al. *JAMA Network Open*.

eFigure 2. River Plot.

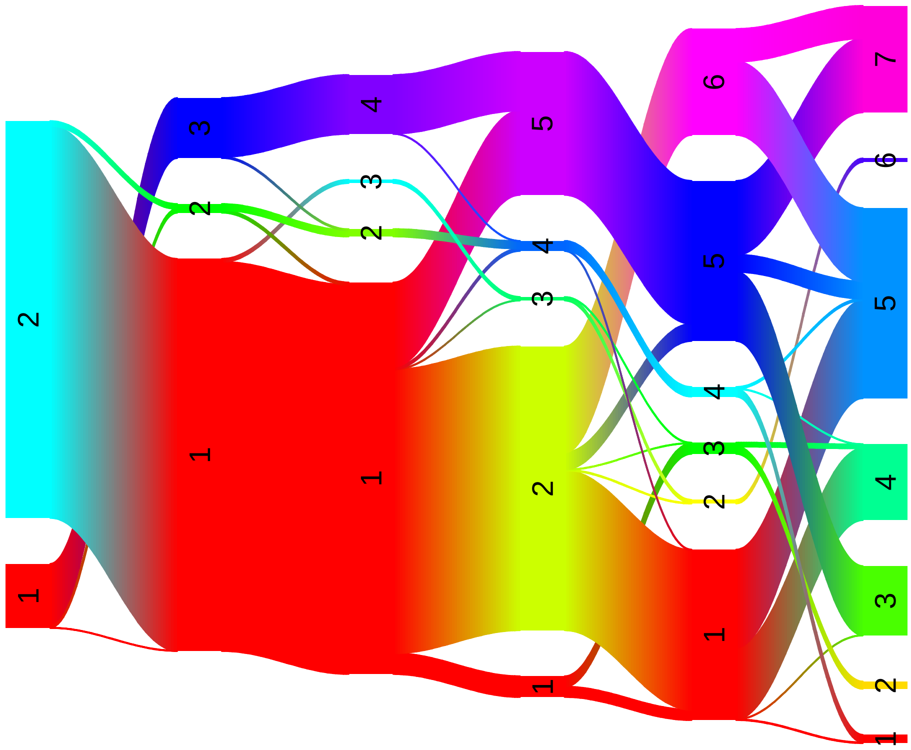

© 2022 Wickwire EM et al. *JAMA Network Open*.
