# Supplementary material for: Trajectories of Insomnia in Adults After Traumatic Brain Injury
Source: JAMA Netw Open. 2022 Jan 26;5(1):e2145310. doi: 10.1001/jamanetworkopen.2021.45310 (PMC8792888; doi:10.1001/jamanetworkopen.2021.45310)
Supplement: Supplement 2. — Nonauthor Collaborators [file jamanetwopen-e2145310-s002.pdf]

Supplemental Online Content: Nonauthor Collaborators

*Indicates required information. Only first name, last name, and suffix will appear in PubMed.

| ***Group Name(s): TRACK-TBI Investigators** | | | | | | | |
|---|---|---|---|---|---|---|---|
| ***First Name and Middle Initial(s)** | ***Last Name** | ***Suffix (eg, Jr, III)** | Academic Degrees | Institution | Location (city, state/province, country) | Role or Contribution, eg, chair, principal investigator | Group (if more than 1 Group listed in the byline) and/or Subgroup (eg, Steering Committee) |
| Ramon | Diaz-Arrastia | | MD PhD | University of Pennsylvania | Philadelphia, PA, USA | Principal Investigator | |
| Neeraj | Badjatia | | MD | University of Maryland, Baltimore | Baltimore, MD, USA | Principal Investigator | |
| Ann-Christine | Duhaime | | MD | MassGeneral Hospital for Children | Boston, MA, USA | Principal Investigator | |
| Shankar | Gopinath | | MD | Baylor College of Medicine | Houston, TX, USA | Principal Investigator | |
| Rao | Gullapalli | | PhD | University of Maryland | Baltimore, MD, USA | Principal Investigator | |
| C. Dirk | Keene | | MD PhD | University of Washington | Seattle, WA, USA | Principal Investigator | |
| Frederick | Korley | | MD PhD | University of Michigan | Ann Arbor, MI, USA | Principal Investigator | |
| Laura | Ngwenya | | PhD | University of Cincinnati | Cincinnati, OH, USA | Principal Investigator | |
| Michael | McCrea | | PhD | Medical College of Wisconsin | Milwaukee, WI, USA | Principal Investigator | |
| Randall | Merchant | | MD | Virginia Commonwealth University | Richmond, VA, USA | Principal Investigator | |
| David | Okonkwo | | MD PhD | University of Pittsburgh | Pittsburgh, PA, USA | Principal Investigator | |
| Claudia | Robertson | | MD | Baylor College of Medicine | Houston, TX, USA | Principal Investigator | |
| Sabrina | Taylor | | PhD | University of California, San Francisco | San Francisco, CA, USA | Clinical Trial Manager | |
| David | Schnyer | | PhD | UT Austin | Austin, TX, USA | Principal Investigator | |

Supplemental Online Content: Nonauthor Collaborators

*Indicates required information. Only first name, last name, and suffix will appear in PubMed.

| ***First Name and Middle Initial(s)** | ***Last Name** | ***Suffix (eg, Jr, III)** | Academic Degrees | Institution | Location (city, state/province, country) | Role or Contribution, eg, chair, principal investigator | Group (if more than 1 Group listed in the byline) and/or Subgroup (eg, Steering Committee) |
|---|---|---|---|---|---|---|---|
| Nancy | Temkin | | PhD | University of Washington | Seattle, WA, USA | Principal Investigator | |
| John | Yue | | MD | University of California, San Francisco | San Francisco, CA, USA | Principal Investigator | |
| Esther | Yuh | | MD PhD | University of California, San Francisco | San Francisco, CA, USA | Principal Investigator | |
| Ross | Zafonte | | DO | Harvard Medical School | Boston, MA, USA | Principal Investigator | |
